# Dengue virus-pandemic influenza virus co-infection results in enhanced influenza virus replication through inhibition of apoptosis

**DOI:** 10.1186/1742-4690-9-S1-O10

**Published:** 2012-05-25

**Authors:** Thomas G Voss, Mei-Chun Chen, Gena J Nichols, Somanna K Naveen, Benjamin T Bradley, Robert W Cross

**Affiliations:** 1 Tulane University, School of Medicine, New Orleans, USA

## Introduction

Dengue fever and Dengue Hemorrhagic fever in humans are the result of infection with Dengue virus, a mosquito-borne member of the Flaviviridae. In 2009, the appearance of a novel, swine origin, pandemic (H1N1) influenza A virus in humans resulted in identification of patients co-infected with dengue and influenza with enhanced clinical disease. To elucidate potential mechanism(s) of enhanced pathogenesis observed during human Dengue/Influenza co-infection, we examined the effects of co-infection in cells (A549) and also in animals.

## Materials and methods

A549 cells were infected with Dengue virus or pandemic influenza A virus. Virus replication was measured by plaque assay and immune fluorescence. Apoptosis was measured by multiple assays including TUNEL, Annexin V expression, and Casapse 3 expression. Ferrets were challenged with Dengue for 24 hours prior to influenza challenge. Clinical disease was monitored for 10 days post Dengue challenge. Nasal aspirates were collected and tissues harvested for virology, immunology, and histology studies through the 10-day in life period. Clinical chemistry and hematology were also measured on infected ferrets.

## Results

In A549, co-infection enhances influenza virus replication and reduces dengue virus replication compared to singly infected A549. Influenza-specific inhibition of dengue replication was dependant on multiplicity of infection (moi) and timing of influenza infection with dengue inhibition detected when influenza infection occurs up to 48 hours post Dengue infection. Co-infected cell apoptosis was reduced suggesting a mechanism for increased influenza virus loads. In ferrets co-infected with dengue and pandemic influenza, influenza virus loads and clinical disease signs compared to influenza infection alone were observed. In the lungs of co-infected ferrets, apoptosis was reduced, confirming in vitro results. Our results indicate a potential pathogenic interaction between dengue and influenza viruses that models human co-infection cases.

